# Searching for synergy: combining systemic daptomycin treatment with localised phage therapy for the treatment of experimental pneumonia due to MRSA

**DOI:** 10.1186/s13104-021-05796-1

**Published:** 2021-09-27

**Authors:** Luca G. Valente, Lea Federer, Manuela Iten, Denis Grandgirard, Stephen L. Leib, Stephan M. Jakob, Matthias Haenggi, David R. Cameron, Yok-Ai Que, Josef Prazak

**Affiliations:** 1 Department of Intensive Care Medicine, Inselspital, Bern University Hospital, University of Bern, 3010 Bern, Switzerland; 2 Institute for Infectious Diseases, University of Bern, Bern, Switzerland; 3 Graduate School for Cellular and Biomedical Sciences, University of Bern, Bern, Switzerland; 4 Department of Biomedical Research, University of Bern, Bern, Switzerland

**Keywords:** Ventilator-associated pneumonia, Nebulized bacteriophages, Methicillin-resistant *Staphylococcus aureus*, Phage-antibiotic interactions

## Abstract

**Objective:**

Bacteriophages (or phages) are viruses which infect and lyse bacteria. The therapeutic use of phages (phage therapy) has regained attention in the last decades as an alternative strategy to treat infections caused by antimicrobial-resistant bacteria. In clinical settings it is most likely that phages are administered adjunct to antibiotics. For successful phage therapy it is therefore crucial to investigate different phage-antibiotic combinations in vivo*.* This study aimed to elucidate the combinatorial effects of systemic daptomycin and nebulised bacteriophages for the treatment of experimental pneumonia due to methicillin-resistant *Staphylococcus aureus* (MRSA).

**Results:**

Using a rat model of ventilator-associated pneumonia caused by MRSA, the simultaneous application of intravenous daptomycin and nebulised phages was not superior to aerophage therapy alone at improving animal survival (55% vs. 50%), or reducing bacterial burdens in the lungs, or spleen. Thus, this combination does not seem to be of benefit for use in patients with MRSA pneumonia.

## Introduction

Ventilator-associated pneumonia (VAP) is a frequent complication for mechanically ventilated patients in intensive care units [1]. The problem of VAP has been compounded by the spread of antibiotic-resistant bacterial strains that respond poorly to standard-of-care therapies. Alternative strategies for the treatment of VAP warrant investigation. In this context, the use of bacterial viruses (called bacteriophages or phages) for the treatment of pneumonia due to methicillin-resistant *Staphylococcus aureus* (MRSA) has been investigated using an experimental rat model designed to mimic VAP [2–4]. Phages were as effective as antibiotics (the glycopeptide teicoplanin) when applied intravenously (IV), each rescuing about half of the animals from severe pneumonia [2]. An ongoing challenge for phage therapy moving forward is the identification and exploitation of phage-antibiotic synergies [5]; however, in the rat VAP model, the IV phage-antibiotic combination did not synergize and still half of the animals treated with the combination succumbed to the infection [2]. To determine if altering pharmacokinetic/pharmacodynamic (PK/PD) parameters could improve the efficacy of phage therapy, phages were nebulized and administered directly to the lungs of MRSA infected rats (‘aerophages’). Aerophages were as effective as the IV phage treatment (50% survival), and the combination of locally administered aerophages and systemically administered IV phages synergized to significantly improve animal survival (> 90%) and reduce lung bacterial burdens when compared to either therapy alone [4]. We reasoned that the efficacy of aerophage monotherapy was limited once the bacterial infection disseminated, and that the combination therapy with systemic phages was most effective as it could combat both the pneumonia and subsequent bacteraemia. Using this rational, replacing IV phages with the frontline antibiotic for MRSA, linezolid, which is likely to be a more clinically acceptable approach, should be similarly effective. This, however, was not observed, and post-hoc analysis of the linezolid-phage combination revealed antagonisms in vitro, whereby linezolid (a bacteriostatic protein synthesis inhibitor) appeared to inhibit phage replication, which was mirrored in vivo [4].

## Main text

### Rationale

In the current report, we tested the combinatorial effects of systemic daptomycin and aerophages for the treatment of experimental pneumonia due to MRSA. Daptomycin was chosen for three reasons. First, it is a bactericidal lipopeptide antibiotic that disrupts the bacterial cell membrane [6], thus it has a mode of action that is distinct from the other antibiotics that have been tested in combination with phages in this model (glycopeptides, oxazolidinone) [2, 4]. Secondly, the daptomycin-phage combination revealed neither synergisms nor antagonisms in vitro, as determined using checkerboard assays (methods described in [2], and finally, daptomycin is not recommended for use for bronchial–alveolar pneumonia as its antibacterial effects are mitigated in the lung by surfactant, yet it has shown efficacy in the context of hematogenous pneumonia [7]. The final two points are of particular importance as they allow us to test the hypothesis that a localised aerophage therapy can be improved in combination with systemic antibiotics; we do not expect improved efficacy due to classical phage-antibiotic synergy, rather the antibiotic effects should be limited to combating bacteria that have spread to sites other than the lung.

### Material and methods

Experiments were performed according to the Committee on Animal Experiments of the Canton of Bern, Switzerland approval BE83/17 and according to ARRIVE Guidelines. Detailed methods about the model were published previously [2–4]. Briefly, a total number of 14 male Wistar rats (Crl:WI(Han), 9–10 weeks old, Charles River, Germany) were ventilated for 4 h (10 mL/kg tidal volume, 5 cm H_2_O positive end-expiratory pressure, 50 breaths per minute with FiO_2_ 0.35) prior to inoculation with alpha-toxin producing MRSA clinical strain AW7 (~ 10^10^ colony forming units (CFU) per animal via the endotracheal tube) [8]. Animals were treated two hours after infection, and again at 12, 24, 48, and 72 h. Treatment was administered in an investigator/operator blinded manner and blinding was maintained until the end of data analysis. Animals were allocated randomly (using GraphPad Prism, v7) to receive either IV daptomycin (6 mg/kg) and a nebulized placebo (filtered supernatant from the bacterial strain used for the phage propagation, n = 3 animals), or IV daptomycin and aerosolized phages (6 mg/kg daptomycin and ~ 2 × 10^10^ plaque forming units (PFU) of a nebulized cocktail of phage K, 3A, 2002 and 2003, n = 11 animals). This specific phage cocktail has been applied previously [2–4]. Phage K (Accession Number AY176327.1) and phage 3A (Accession Number NC_007053.1) were purchased from the University of Laval, Québec. Phage 2002 (Accession Number MW528836.1) was isolated from sewage water in Lausanne, Switzerland. Phage 2003 was isolated from the staphylococcal phage product of the Eliava Institute of Bacteriophages, Microbiology and Virology, Tbilissi, Georgia.

**Fig. 1:**
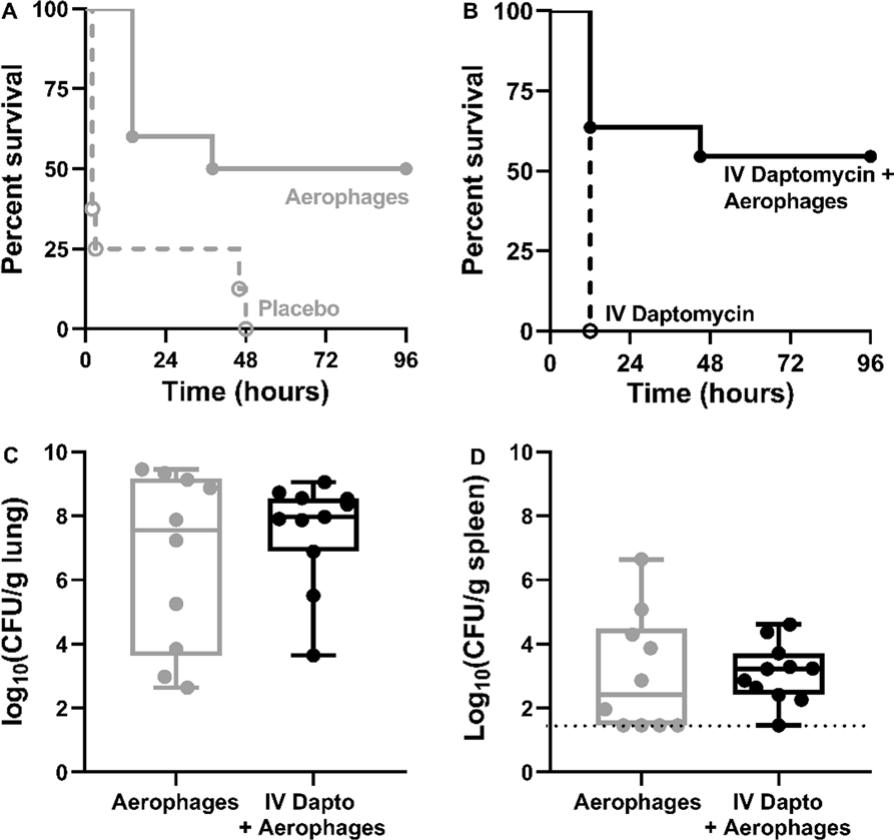
Combination IV daptomycin and aerophages in an experimental model of VAP due to MRSA. **A** Kaplan–Meier survival analysis for rats with pneumonia due to MRSA treated with either placebo (nebulized placebo and IV placebo, n = 8) or aerophages (~ 10^10^ plaque forming units (PFU) of nebulized phage cocktail and IV placebo, n = 10), as reported in a previous study (4). **B** Kaplan–Meier survival analysis for rats with pneumonia due to MRSA from the current study, treated with either IV daptomycin (6 mg/kg and nebulized placebo, n = 3) or IV daptomycin and aerophages (6 mg/kg daptomycin IV, ~ 2 × 10^10^ PFU of nebulized phage cocktail, n = 11). **C** Bacterial densities in the lungs of animals following euthanasia. **D** Bacterial densities in the spleen of animals following euthanasia. The dotted line represented the limit of detection. Dapto, daptomycin; CFU, colony forming unit

Based on our previous studies, the aerophage therapy alone results in 50% survival (Fig. 1A) [4]. We hypothesized, that the combination of aerophage treatment with IV daptomycin would increase survival to 95–100%, similar to the combination of IV phages with aerophages [4]. These estimates (alpha = 0.05, power 1−β = 0.8) required n = 11 per group (SigmaPlot 12.0). Animals were monitored for 96 h, and illness was scored as described previously [2]. Animal survival at 96 h was the primary endpoint, and lung and spleen bacterial densities were determined following euthanasia as secondary endpoints. Animals scored as severely ill or reaching the primary endpoint at 96 h were euthanized as a humane endpoint using pentobarbital (150 mg/kg) injected intraperitoneally. Control groups (placebo treated, aerophage treated alone) were published in a previous study [4] and the data is included in the current study for comparison to *reduce* the number of animals required for experimentation according to the 3R framework (Fig. 1A). Survival of animals was assessed using Kaplan–Meier curves and log-rank tests. All analyses were performed using GraphPad Prism (v7).

### Results

Three animals with pneumonia due to MRSA were treated with daptomycin IV plus nebulized placebo and each succumbed to infection within 12 h. The combination of daptomycin IV and aerophages rescued 55% of animals from infection by the end of the 96-h trial (Fig. 1B). The combination, however, was not superior to aerophage therapy alone at improving animal survival (50%, Fig. 1B), or reducing bacterial burdens in the lungs, or spleen (which we use as a marker for systemic spread of infection) (Fig. 1C).

### Discussion

It is likely, particularly in critical care situations, that phage therapy will be administered adjunct to antibiotics [5, 9]. The efficacy of phage therapy in animal models has now been tested extensively, however, only few studies have directly compared the efficacy of phages and antibiotics or assessed the efficacy of antibiotic-phage combinations in vivo. Worryingly, the same antibiotic-phage combination can show either synergisms or antagonisms depending on the concentration of each agent when tested in vitro [10], and the efficacy of a given phage-antibiotic combination can depend on the laboratory method used for assessment [2, 4, 10, 11]. Robust and standardized in vitro methods assessing phage-antibiotic synergies should be developed that can accurately predict improved treatment outcomes in vivo. Additionally, in vitro experiments have shown that staggered administration of phages and antibiotics (one, then the other, as opposed to each at the same time) may be more effective than antibiotics alone for the treatment of MRSA [11], and this warrants further investigation in a model such as that used in the current study. In conclusion, combination IV daptomycin and aerophages does not appear appropriate for use in patients with VAP, thus our search for in vivo phage-antibiotic synergy in MRSA pneumonia continues.

## Limitations

As for most small animal models designed to mimic human infection, the model used in this study has some limitations. First, human VAP is defined as pneumonia occurring 48 h after the start of mechanical ventilation [12]. However, ventilation of rats over long time periods leads to extensive lung damage irrespective of infection and/or adequate treatment, limiting the time of ventilation in the model to 4 h. Second, VAP is usually established when a small inoculum of bacteria reaches the lower respiratory tract, where they adhere to the mucosa and start the infection [13]. In the current model, in order to establish reproducible pulmonary infection in immunocompetent rats, a high bacterial load (~ 10^10^ CFU per animal) had to be applied directly to the lungs via the endotracheal tube. Small reductions of the inoculum resulted in spontaneous clearance of the infection. As a consequence, the high inoculum led to severe infection whereby treatment needed to be administered quickly (2 h after infection) in order to avoid extensive mortality.

## Data Availability

The datasets and material used and/or analysed during the current study are available from the corresponding author on reasonable request.

## Abbreviations

VAP: Ventilator-associated pneumonia
MRSA: Methicillin-resistant *Staphylococcus aureus*
IV: Intravenously
PK/PD: Pharmacokinetic/pharmacodynamic
Aerophages: Aerosolized bacteriophages administered to the lungs
CFU: Colony forming units
PFU: Plaque forming units
